# The Current Landscape of Artificial Intelligence in Imaging for Transcatheter Aortic Valve Replacement

**DOI:** 10.1007/s40134-024-00431-w

**Published:** 2024-10-10

**Authors:** Shawn Sun, Leslie Yeh, Amir Imanzadeh, Soheil Kooraki, Arash Kheradvar, Arash Bedayat

**Affiliations:** 1 Radiology Department, UCI Medical Center, University of California, Irvine, USA; 2 Department of Radiological Sciences, University of California, Los Angeles, CA 90095, USA; 3 Department of Biomedical Engineering, University of California, Irvine, CA 92697, USA; 4 Independent Researcher, Anaheim, CA 92803, USA

**Keywords:** Transcatheter aortic valve replacement (TAVR), Artificial intelligence (AI), Computed tomography (CT)

## Abstract

**Purpose:**

This review explores the current landscape of AI applications in imaging for TAVR, emphasizing the potential and limitations of these tools for (1) automating the image analysis and reporting process, (2) improving procedural planning, and (3) offering additional insight into post-TAVR outcomes. Finally, the direction of future research necessary to bridge these tools towards clinical integration is discussed.

**Recent Findings:**

Transcatheter aortic valve replacement (TAVR) has become a pivotal treatment option for select patients with severe aortic stenosis, and its indication for use continues to broaden. Noninvasive imaging techniques such as CTA and MRA have become routine for patient selection, preprocedural planning, and predicting the risk of complications. As the current methods for pre-TAVR image analysis are labor-intensive and have significant inter-operator variability, experts are looking towards artificial intelligence (AI) as a potential solution.

**Summary:**

AI has the potential to significantly enhance the planning, execution, and post-procedural follow up of TAVR. While AI tools are promising, the irreplaceable value of nuanced clinical judgment by skilled physician teams must not be overlooked. With continued research, collaboration, and careful implementation, AI can become an integral part in imaging for TAVR, ultimately improving patient care and outcomes.

## Introduction

The introduction of transcatheter aortic valve replacement (TAVR) in the 2000s revolutionized the management of severe aortic stenosis (sAS). Multiple randomized controlled trials (RCT) have demonstrated improved perioperative mortality rates and favorable long-term outcomes for patients receiving TAVR [1, 2]. Society guidelines have now established TAVR as a standard of care for a selected group of patients with sAS [3]. The number of TAVR procedures performed in the United States has grown substantially each year, with approximately 98,500 TAVR procedures performed in the US in 2022 [4].

Noninvasive imaging techniques play a crucial role for patient selection and preprocedural planning for TAVR. Multidetector CT and, less commonly, MRA are used for access planning, annular sizing, product selection, and other assessments. Precise anatomical measurements are required for optimal valve selection and predicting the risk of post-TAVR complications such as paravalvular leak, valve migration, and coronary obstruction. In 2019, a consensus was published on the correct technique for performing and analyzing these pre-operative scans for TAVR [5]. However, this complex process remains time-consuming and labor-intensive for radiologists, even with the use of semi-automatic software.

The indication for TAVR has expanded from patients being ineligible or at high-risk for surgical intervention to include patients at intermediate and low risk for surgical aortic valve replacement (SAVR) [3]. Several ongoing clinical trials aim to broaden FDA approval of TAVR for asymptomatic sAS and potentially even moderate AS [6, 7]. The growing demand for TAVR procedures and the necessary pre-operative planning will exacerbate the burden on radiologists who are already grappling with a global shortage [8].

The rise of artificial intelligence (AI) over the past decade has propelled this innovation into society’s mainstream and holds the potential to revolutionize radiology and medicine [9]. Several recent studies have demonstrated AI’s potential to enhance pre-operative planning efficiency in TAVR, promising to further improve patient care. Additionally, fully automated AI tools are currently under development to streamline this process [10].

This review emphasizes the current state-of-the-art research in AI techniques for TAVR-related imaging, addressing their limitations and exploring future directions.

## Patient Selection and Diagnosis

The diagnosis of AS via echocardiography relies on certain operator-dependent measurements, which can affect specific subgroups based on who performs the scan. For example, inter-operator variability for the left ventricular outflow tract (LVOT) diameter reaches up to 8%, which is then magnified in the calculation of aortic valve area (AVA) [11]. Traditionally, a mean pressure gradient of 40 mmHg or greater and an AVA of less than 1 cm^2^ are considered the criteria for diagnosing sAS [3]; however, recent findings suggest that up to one-third of patients present with discordant AS grading [12]. Measurement error and discordant grading severely impact the diagnosis of sAS, and accurate grading is crucial for timely intervention and proper management of these patients.

AI holds promise in reducing misclassification by assisting in the diagnosis and identification of aortic stenosis from echocardiograms. Holste et al. [13] developed a deep learning model that detects sAS from 2D cine echocardiography data, eliminating the need for Doppler imaging. This model was trained and validated on large datasets from multiple institutions, achieving high diagnostic accuracy for aortic stenosis, with AUC of 0.978 in the primary test set. A model using only 2D single views has the potential for point-of-care screening for sAS across various clinical settings and patient subtypes. Playford et al. [14] explored the use of an AI algorithm to identify sAS from routine echocardiograms without the need for error-prone LVOT information. Key results show that the algorithm could correctly identify 95.3% of patients with traditional high-gradient AS and 100% of patients with traditional high-gradient AS and AVA < 1.0 cm^2^. It also effectively identified low-flow, low-gradient severe AS, with only a 4.7% misclassification rate.

While echocardiography remains the gold standard for diagnosing and grading AS, its use as a routine screening method may be impractical due to the significant costs, required operator skill, and time involved. As a result, there is a growing interest in more cost-effective and widely accessible solutions, such as the use of ECGs and chest radiographs [15, 16] for early detection of AS. Elias et al. developed a deep learning model, ValveNet, to detect left-sided valvular heart diseases using ECG data [15]. This model achieved high diagnostic accuracy, with an AUC of 0.84 for detecting AS, aortic regurgitation (AR), or mitral regurgitation (MR), and demonstrated a sensitivity of 0.78 and a specificity of 0.73. When optimized for screening, the model achieved a positive predictive value (PPV) of 0.20 and negative predictive value (NPV) of 0.98 at a prevalence of 7.8%. These findings highlight the potential of ValveNet in detecting valvular heart conditions and for use as a screening tool. Ueda et al. developed and evaluated three deep learning models to detect AS from chest radiographs using a dataset of 10,433 images from 5638 patients [16]. These radiographs were classified based on echocardiography assessments as either AS-positive or AS-negative. The top-performing model combined weighted averages of probability scores from all three individual models, achieving an AUC of 0.83, sensitivity of 0.78, specificity of 0.71, accuracy of 0.71, PPV of 0.18, and NPV of 0.97 on the validation dataset. The integration of AI with chest radiographs and ECGs holds great promise for enhancing AS detection as a highly efficient screening tool.

Language models were also studied for identifying patients at risk of AS through electronic medical record (EMR) documentation. Solomon et al. developed and validated natural language processing (NLP) algorithms to accurately detect patients with AS from echocardiogram reports [17]. Using 1003 physician-adjudicated reports, NLP algorithms achieved a PPV and NPV over 0.95 for AS detection. Applying these algorithms to nearly one million echocardiograms revealed that NLP identified AS in 11.2% of cases, demonstrating superior accuracy compared to diagnosis codes. This underscores the potential of NLP to improve AS identification and management within healthcare systems.

## Pre-procedural Planning for TAVR

Pre-procedural CTA or MR for TAVR focuses on several specific tasks: (1) assessment of the aortic annulus and root for selection of the prosthesis, (2) assessment of the supravalvular aorta and vascular access to map the device delivery path, (3) risk stratification for annular injury or coronary occlusion, and 4) prediction of the fluoroscopic angle for valve deployment [5, 18]. More specifically, pre-procedural planning typically includes an evaluation of the options for endovascular access, identification of the annular plane with precise measurements of the annulus, bicuspid versus trileaflet valvular morphology, presence of aortic root calcifications, relationship of the coronary ostia to the native valve leaflets, angulation between the ascending aorta and the LVOT, and possible incidental findings [19]. Further details regarding the technique can be found in the published consensus statement [5].

In addition to being time-consuming and labor-intensive, manual analysis of pre-TAVR imaging entails significant inter-operator variability [20], which can potentially impact the procedural planning and increase the risk of procedure-related complications [21]. AI algorithms developed to automate this process show promise in improving standardization, reducing provider workload, and lowering overall healthcare costs [10], [22] (First available AI based method to evaluate TAVR measurements), [23, 24], [25] (First to obtained FDA clearance and CMS billing code), [26-29].

### TAVI-PREP

In 2023, Santaló-Corcoy et al. introduced TAVI-PREP, one of the first fully automated deep learning-based tools for extracting the full ensemble of critical measurements from pre-TAVR planning CT scans [22] (First available AI based method to evaluate TAVR measurements). Their tool incorporates a deep learning-based segmentation algorithm and landmark detection algorithm that creates a 3D mesh representing the patient’s cardiac and aortic anatomy, from which measurements are then derived. It requires approximately 2 min to extract 22 measurements, including measurements of the annulus, LVOT, sinotubular junction (STJ), sinus of Valsalva (SOV) commissures, and the coronary artery heights. The authors then compared the results of their automated tool with two expert cardiologists on a dataset of 200 patients. High correlation coefficients (CC) were obtained for most measurements between the algorithm and the expert measurements, ranging from 0.9 to 0.97.

Nevertheless, the algorithm struggled with predicting coronary ostia heights, with CCs of 0.72 to 0.80 in addition to several other limitations. Lower CCs were found between the algorithm and the expert readers for prediction of coronary artery heights. Edge cases, including patients with severe calcification or artifacts present on the CT images, posed challenges to the algorithm, and led to less accurate predictions. Valve selection, aortic angle, and femoral access diameters were also not included in the algorithm at the time of publication. Moreover, one of the expert readers used in the comparison provided the annotations for training the software, and subsequently had a higher correlation with the software compared to the other expert, which is speculative of bias. Incorporation of additional measurements, improvement of coronary artery height prediction, and performing additional external validation studies may strengthen the argument for clinical integration readiness.

### MIMICS PLANNER^™^

The Mimics Planner^™^ software tool (Materialise, Leuven, Belgium) automates preprocedural planning for structural heart disease. This tool utilizes machine learning for heart segmentation to obtain accurate measurements and visualizations of anatomy in 3D. Mimics Planner^™^ has been used for aortic, mitral, and tricuspid valve implantation and left atrial appendage occlusion planning [23].

Due to this product’s proprietary nature and lack of published data, details of the Mimics Planner^™^ tool’s technology are limited. A validation study was performed by Corbin et al. to compare the Mimics Planner^™^ tool with the TAVI-PREP algorithm on the same 200 patients in the original TAVI-PREP study [24]. Overall, TAVI-PREP achieved greater performance on perimeter-associated measurements and Mimics Planner^™^ achieved more accurate predictions of sinus measurements. Both algorithms struggled with coronary height measurements.

Although further details about the Mimics Planner^™^ are lacking, advantages include additional functions beyond anatomical measurements including device selection, 3D visualization, valvular calcification scoring, and report preparation.

### Precision TAVI

Precision TAVI^™^ (Dasi Simulations, Dublin, Ohio) obtained FDA approval in 2023 and even a CMS outpatient billing code in 2024 for preprocedural TAVR planning. Their software utilizes patient specific CTA imaging to perform simulation testing; however, there is no published data on the efficacy of the overall product. There are several published studies demonstrating individual techniques, including using preprocedural data points to predict post-TAVR aortic valve pressure gradient and aortic valve area size [25] (First to obtained FDA clearance and CMS billing code), predicting post-TAVR valve thrombosis [26], and predicting the risk of coronary obstruction post-TAVR through measurement of coronary artery heights [27, 28].

### 4TAVR

Toggweiler et al. introduced 4TAVR in 2024, a fully automated software tool, which analyzes patient CTAs, performs anatomical segmentation and 3D reconstruction, calculates planning measurements, and ultimately generates reports in approximately ten minutes [29]. This tool is now available for use through the Hi-D Imaging Cardiovascular Imaging Suite (Hi-D Imaging, Winterthur, Switzerland). 4TAVR utilizes 2D and 3D U-net models for segmentation and a separate U-net model for landmark detection. Automated multiplanar reconstructed slices are then created based on the extracted aortic centerline for anatomical measurements. The results of the automated measurements were compared with three expert operators in 100 TAVR patients at a single center. The tool achieved a CC of up to 0.97 for the annular perimeter and area and 0.83–0.95 for measurements of the LVOT, SOV, STJ, and ascending aorta. Advantages of the study were the inclusion of cases with heavy calcification burden and bicuspid valves. In addition, AI-generated measurements resulted in > 85% agreement with manual measurements in valve sizing for multiple types of valves. The major limitation of this study is the small patient cohort from a single center.

### Philips HeartNavigator

HeartNavigator by Philips (Philips, Amsterdam, Netherlands) is a fully automated software analysis of pre-procedural CTA that offers segmentation and measurement of the heart anatomy, e.g., the annulus, SOV, and coronary artery heights. Koĉka et al. prospectively evaluated the tool’s capabilities in 128 patients at a single center in Prague versus manual analysis using FluoroCT software performed by lab technicians supervised by a cardiologist [30]. Overall, they found a statistically significant difference between the manual and automatic measurements though the numerical difference was small; more specifically, less than 2 mm on average for most measurements. Furthermore, the manual and automatic measurements yielded the same valve size selection for the Evolut PRO valves in 80% of cases. Another advantage is that the CT analysis can be utilized for fusion imaging with fluoroscopy during the procedure.

Additional studies involving a larger patient cohort, multi-center participation, comparison with radiologist measurements, and evaluation with different types of valves would be necessary for further validation.

### FORSSMAN

Wang et al. developed FORSSMAN, a fully automated deep learning-based tool for aortic valvular complex segmentation and measurement [31]. This tool employs a U-Net-based two-stage deep learning network for segmentation and landmark detection. It performs centerline extraction and generates key planes, from which measurements are derived. In addition to measurements of the annulus and SOV, the coronary heights, aortic angle and volume of calcification are extracted. The FORSSMAN tool was evaluated on an external validation dataset of 100 patients from over 19 hospitals in China and the automatic measurements were compared to manual measurements by 2 senior observers. On average, the automated tool required 0.9 min for measurement extraction compared to 19.5 min for manual measurements. Comparison with senior observers resulted in CCs of greater than 0.97 for most measurements, with only the aortic angle having a CC of 0.87.

Advantages of this model include testing on edge cases, such as severe valvular calcification and bicuspid valves, and automatic detection of anatomical risk factors, including horizontal aorta, ascending aorta dilation, severe calcification, large SOV size, and low coronary ostia height. Limitations of this study mostly include the small patient cohort, lack of information on the impact of automatic versus manual measurements on overall valve selection, and lack of evaluation of the access route.

### Vascular Module of AI-RAD Companion Chest CT

Boninsegna et al. evaluated a Vascular Module Platform developed by Siemens Healthineers (Erlangen, Germany) on 50 patients prior to TAVR [32]. This tool utilizes an adversarial deep image-to-image network in a symmetric convolutional encoder-decoder architecture to perform segmentation of the cardiac anatomy. A centerline model is then combined with landmark detection to identify measurement planes. The Vascular Module automatically extracts the diameters of the aorta at 9 positions, including the SOV, STJ, and points along the ascending, arch, and descending portions of aorta. On average, the AI measurements required 1 min and 47 s whereas manual measurements took 5 min and 41 s. Overall, the AI- and manual-obtained values were not significantly different, and for 91% of values the difference was ≤ 1 mm.

**Fig. 1:**
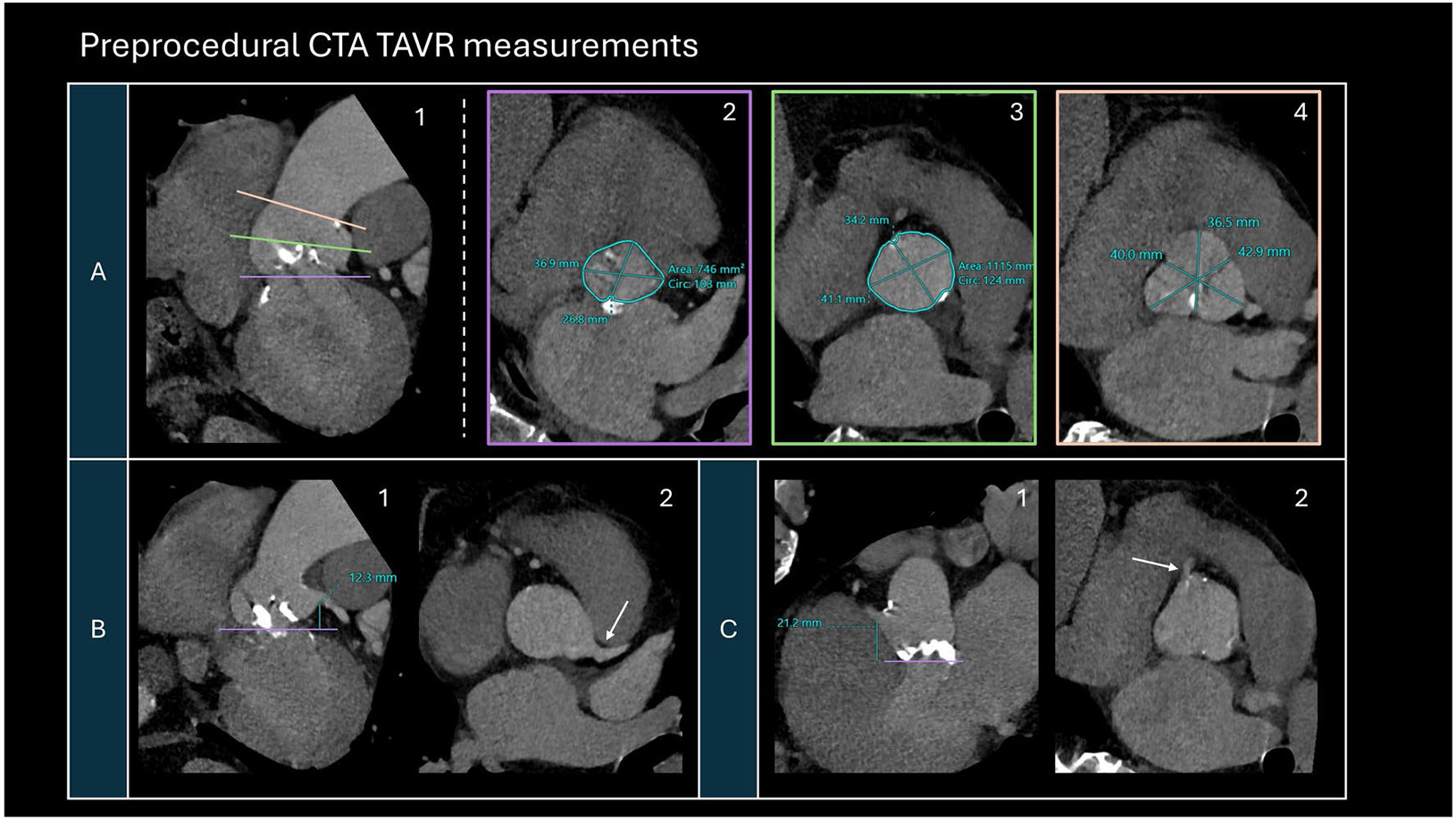
A representative sample of common preprocedural CTA TAVR measurements commonly performed for operative planning and valve selection. **A** Multiplanar double oblique images of the aortic root along the long axis (A1) with levels (solid lines) corresponding to the true short axis views of the aortic annulus (A2), sinotubular junction (A3), and Sinuses of Valsalva (A4). **B** Measurement of the coronary ostia height of the left main coronary artery at the takeoff from the left coronary cusp (arrow) as seen on the longitudinal (B1) and the short axis (B2) views. **C** Measurement of the coronary ostia height of the right coronary artery at the takeoff from the right coronary cusp (arrow) as seen on the longitudinal (C1) and the short axis (C2) views

Limitations of this tool include the lack of aortic annulus measurement, assessment of the access route, consideration of additional factors such as bicuspid valves or valvular calcifications, and the small sample size at a single institution (see Fig. 1).

## Coronary Artery Screening

Patients with sAS commonly have multiple cardiovascular comorbidities, such as coronary artery disease (CAD), which can lead to ischemia following hemodynamic instability during TAVR procedures [33]. Although invasive coronary angiogram has previously been the gold standard for CAD evaluation, concomitant evaluation of the coronary arteries during the pre-procedural CTA has been shown to be safe and effective and is now routinely used for CAD screening prior to TAVR in select populations [3, 34]. Currently, coronary CTA (CCTA) has moved beyond assessment of luminal stenosis to also involve characterization and quantification of atherosclerosis. Several semi-automated software tools can provide measurements of plaque volume and composition; however, the analysis is time-consuming and demands significant manual input from expert readers.

In a multicenter study, Lin et al. evaluated a novel deep learning system for accurate quantification of plaque volume and stenosis severity from CCTA [35]. Automated measurements achieved intraclass CCs of 0.96 for plaque volume and 0.88 for stenosis severity when compared to expert readers. AI analysis required an average of 5.7 s, compared to the 25.7 min typically required by expert readers for manual measurements. This model was constrained by semi-automated coronary centerline extraction performed by technologists and validation in a small sample size of patients.

In another multicenter study, Choi et al. evaluated the FDA-cleared software Cleerly LABS (Cleerly, New York, New York) for automated analysis of CCTA for comprehensive CAD assessment [36]. Key measurements including percent stenosis, plaque volume and composition, presence of high-risk plaque, and CAD-RADS category were compared between the AI algorithm and expert readers. Correlation was best with detecting percent stenosis, with intraclass CCs of 0.91 for per-vessel and 0.93 for per-patient evaluation between AI and expert analyses. Greater than 98% agreement was found for determination of CADS-RADS categories on a per-patient evaluation. AI analysis alone required an average of 9.7 min, and when including quality assurance and report generation, it totaled an average of 23.7 min. The time required for expert readers per case was not assessed.

Coronary artery calcium scoring, typically assessed by the Agatston score, has demonstrated prognostic value in predicting mortality among patients undergoing TAVR [37]. Several machine learning algorithms have shown high performance in fully automating coronary artery calcium scoring [38].

CT angiography-derived fractional flow reserve (CT-FFR) assessment has been shown to enhance the prediction of high-risk patients compared to CTA alone, but accessibility and computational time have been limiting factors thus far [39]. Recent advancements in CT-FFR have introduced machine learning algorithm-based acquisition methods, replacing the previous standard of computational fluid dynamics. Current data suggests that machine learning-derived CT-FFR improves diagnostic performance compared to CCTA alone [40].

## Outcome Prediction Post-TAVR

### Post-TAVR Mortality

Mortality post-TAVR has been shown to be comparable to that of post-SAVR, with the 1-year mortality or disabling stroke rate estimated at 1.0% for low-risk patients [41]. Initial efforts to estimate the risk of TAVR led to in-hospital mortality and stroke risk calculators developed from the STS/ACC TVT Registry data [42, 43]. These calculators utilized covariates selected from a combination of expert opinion and logistic regression analysis, and primarily utilized clinical and operative technique-related factors. Recent studies have sought to improve these risk calculators with machine learning models [44] and have demonstrated additional performance in predicting in-hospital mortality for TAVR.

Additionally, preprocedural imaging findings have shown predictive capabilities for post-TAVR mortality, independent of the clinical risk factors utilized in the STS/ACC TVT calculators [45]. Hossain et al. found that pericardial effusions and increased size of the main pulmonary artery, both signs of right heart failure, were predictors of mortality at 1-year post-TAVR. This was also corroborated by the association of decreased right ventricular ejection fraction on echocardiography with mortality at 1-year post-TAVR. Another study by Aquino et al. also showed that left atrial emptying fraction on preprocedural cardiac CTA predicted mortality in patients with severe AS undergoing TAVR [46].

As the radiological assessment of preprocedural imaging is a time-consuming process, Brüggemann et al. developed a 3D deep neural network-based model to automatically predict post-TAVR mortality using unprocessed, preprocedural CT and additional clinical characteristics [47] (First AI application for patient selection). This model outper-formed the models using solely clinical factors and were similar in performance to models using both clinical factors and manually extracted image measurements. Furthermore, while manual extraction required 10 to 15 min for their expert radiologists, the AI model only required 5 to 20 s on a consumer CPU.

### Computational Modeling of Fluid Dynamics

Computational modeling for simulating hemodynamics and tissue behavior is another technique with potential to predict postoperative complications. This approach has been used to “virtually” implant valves for testing of multiple device sizes and implantation depths [48] to determine the optimal selection. However, these models require lengthy computation times and are not routinely used due to practicality and availability constraints. Techniques using machine learning or deep learning have been shown to cut down computation times by an order of magnitude with similar results to conventional methods [49].

The FEops HEARTguide^™^ (FEops, Gent, Belgium) is one such software that employs patient’s CT scans for 3D visualization and semi-automatic identification and measurement of anatomical landmarks to perform patient-specific simulations of valve implantation. The PRECISE-TAVI study, a prospective multicenter observational study, used this software to predict the risk of conduction abnormalities and the risk of paravalvular leakage post-TAVR [50]. This led to changes in procedural planning in 35% of patients, including changes in valve size selection and implantation depth.

## Conclusion

The integration of AI into imaging for TAVR represents a promising advancement in the fields of cardiology and radiology, offering the potential to streamline pre- and post-TAVR care. The ability of AI tools to fully automate and standardize patient identification and selection, pre-procedural imaging analysis, and prediction of post-TAVR mortality would have a significant impact on patient care. Preliminary studies of these AI tools show promise, but these tools are often constrained by small patient cohorts, limited scope, and significant variability in function and performance. Collaboration among AI developers, radiologists, and cardiologists is crucial to ensure these technologies are effectively integrated into clinical practice. Before widespread clinical adoption, rigorous validation will be necessary to ensure the performance of these tools and their overall impact on patient outcomes. Further studies, head-to-head comparisons, and cost–benefit analyses proving reduction in labor or reading time will be necessary to justify the added cost for these programs prior to clinical integration. With continued research, collaboration, and careful implementation, AI can become an integral part in imaging for TAVR, ultimately improving patient care and outcomes.

## Data Availability

No datasets were generated or analysed during the current study.
